# MYCN mRNA degradation and cancer suppression by a selective small-molecule inhibitor in MYCN-amplified neuroblastoma

**DOI:** 10.3389/fonc.2022.1058726

**Published:** 2022-11-24

**Authors:** Tao Liu, Lubing Gu, Zhongzhi Wu, Najah Albadari, Wei Li, Muxiang Zhou

**Affiliations:** ^1^ Department of Pediatrics and Aflac Cancer and Blood Disorders Center, Emory University School of Medicine, Atlanta, GA, United States; ^2^ Department of Pharmaceutical Sciences, College of Pharmacy, University of Tennessee Health Science Center, Memphis, TN, United States

**Keywords:** MYCN, MDM2, small-molecule inhibitor, neuroblastoma, cancer

## Abstract

Amplification of the *MYCN* gene leads to its overexpression at both the mRNA and protein levels. Overexpression of *MYCN* mRNA may also have an important role in promoting neuroblastoma (NB) beyond the translation of MYCN protein. In the present study, we report a small molecule compound (MX25-1) that was able to bind to the 3’UTR of *MYCN* mRNA and induce *MYCN* mRNA degradation; this resulted in potent cell-growth inhibition and cell death specifically in *MYCN*-amplified or *MYCN* 3’UTR overexpressing NB cells. To evaluate the role of *MYCN* 3’UTR-mediated signals in contributing to the anticancer activity of MX25-1, we examined the status and activation of the tumor suppressor microRNA (miRNA) let-7, which is a target of *MYCN* 3’UTR in *MYCN*-amplified NB. We first observed that overexpression of *MYCN* mRNA was associated with high-level expression of the let-7 oncogenic targets DICER1, ARID3B and HMGA2. Following *MYCN* mRNA degradation, the expression of DICER1, ARID3B and HMGA2 was downregulated in MX25-1-treated cells. Inhibition of let-7 reversed the downregulation of these oncogenic mRNAs and significantly increased resistance of NB cells to MX25-1. Our results from this study supported the notion that overexpression of *MYCN* mRNA due to gene amplification has an independent function in NB cell growth and disease progression and suggest that targeting *MYCN* mRNA may represent an attractive strategy for therapy of *MYCN* amplified NB, both by inhibiting MYCN’s cell-survival effects and activating the tumor-suppressor effect of let-7.

## Introduction

NB is the most common extracranial solid tumor in children. Approximately half of all patients with NB are diagnosed with high-risk disease, which is associated with an overall survival rate of less than 50% (1). An important factor predicting poor prognosis is the amplification of the *MYCN* gene, which occurs in 30-40% of all high-risk NB tumors; this amplification strongly correlates with advanced-stage disease and treatment failure (2). Not surprisingly, MYCN has emerged as a target for individualized therapy of *MYCN*-amplified NB. However, despite decades of research in MYCN biology, inhibition of MYCN as a therapeutic strategy in *MYCN*-amplified NB remains an elusive goal.

Much of our previous understanding of the biological activity of MYCN comes from studies focusing on MYCN protein, which acts as a transcriptional factor and plays a critical role in controlling diverse aspects of cellular physiology. There are hundreds of proposed candidate target genes of MYCN. In *MYCN*-amplified NB, overexpressed MYCN protein regulates its oncogenic targets; these include MDM2 and genes of the *PI3k/Akt/mTOR* and *STAT* signaling pathways, which have anti-apoptotic and growth-promoting effects for NB cells (3, 4). However, high levels of MYCN protein in *MYCN*-amplified NB can also sensitize tumor cells to drug-induced apoptosis by transactivating tumor suppressor targets such as p53 (5). In addition, the structure of MYCN is composed almost entirely of α helices with no surface for ligand binding and molecular targeting. Due to the complex functions of MYCN protein and the difficulty for targeting, attempts to selectively inhibit MYCN protein for therapy of *MYCN*-amplified NB tumors have met with little success (6).

Recent studies showed that *MYCN* mRNA also has an important role in NB beyond the translation of MYCN protein. Overexpression of *MYCN* mRNA in *MYCN*-amplified NB has an independent function promoting NB cell proliferation through inhibiting the tumor suppressor miRNA let-7: the 3’UTR of *MYCN* mRNA has multiple binding sites for let-7, and binding of let-7 to *MYCN* 3’UTR can inhibit let-7 activity (7). Let-7 is a small non-coding RNA molecule that serves as a potent tumor suppressor *via* post-transcriptional repression of multiple oncogenic mRNA targets such as DICR1, ARID3B and HMGA2 (8–10). It has been shown that let-7 plays an important tumor-suppressor role in NB cells by inhibiting NB cell proliferation (11). Previous studies demonstrated that suppression of let-7 by Lin28B or loss of let-7 expression (resulting from chromosomal 11q or 3p deletion) in NB cells is correlated with poor-prognosis (11, 12).

Apart from gene amplification, the expression of *MYCN* mRNA is also regulated by other cellular signals that modulate *MYCN* mRNA stability. For example, the stability of *MYCN* mRNA is regulated by the AU-rich elements (ARE) within its 3’UTR, which provide signals for rapid degradation of the mRNA (13). HuD, a neuronal-specific RNA-binding protein, has been shown to bind to the ARE of the *MYCN* 3’UTR and stabilize *MYCN* mRNA (14). Our previous studies identified that MDM2 also binds to the ARE of the *MYCN* 3’UTR and stabilizes *MYCN* mRNA (15).

The MDM2 protein has been well-characterized as an RNA-binding protein (16), and MDM2 is able to bind to many other cellular mRNAs including *XIAP*, *VEGF*, *Slug* and *p53*, in addition to *MYCN*, to regulate their translation in cancer cells (17–20). In contrast, RNA-binding to the C-terminal RING domain of MDM2 can stabilize MDM2 protein; for example, we have found that binding of *XIAP* IRES mRNA to MDM2 stabilizes the protein and leads to increased tumor-cell survival (21). Based on the interaction between MDM2 RING domain and *XIAP* IRES, we have previously developed a fluorescence polarization (FP) assay for high-throughput screening (HTS) of chemical libraries, in order to select small-molecule inhibitors to block the interaction and inhibit expression of both MDM2 and XIAP. Our FP-HTS studies identified a group of small-molecule heterocyclic compounds (including MX25) that bind either to MDM2 protein or to *XIAP* mRNA to block their interaction and inhibit both MDM2 and XIAP expression (22).

During our studies of chemical modifications of these selected compounds to develop potential anticancer drugs, we identified an analog (MX25-1, containing ethyl sulfate as the counter ion to MX25) of one of the most potent *XIAP* mRNA-binding compounds (MX25) that was able to bind to the 3’UTR of *MYCN* mRNA. In the present study, we evaluated whether the binding of MX25-1 to *MYCN* 3’UTR is able to block its interaction with MDM2 and inhibit both *MYCN* mRNA and MDM2 expression, resulting in tumor growth inhibition and cell death. Using *MYCN*-amplified NB cell lines and MX25-1 as a probe, we have particularly investigated the independent function of *MYCN* mRNA, in addition to MYCN protein and MDM2, in promoting NB cell growth. We have also evaluated the effect of *MYCN* mRNA inhibition alone on MX25-1-induced NB growth inhibition and cell death.

## Materials and methods

### Cell lines and treatment

Six human NB cell lines (NB-1643, NB-1691, LA1-55N, SK-N-SH SHEP1 and SHEP/21N/Tet-) were used in this study. Three of the six NB cell lines (NB-1643, NB-1691 and LA1-55N) had *MYCN* gene amplification. SK-N-SH and SHEP1 had no *MYCN* gene amplification and SHEP/21N/Tet- had conditional MYCN expression. Five of the six lines (NB-1643, NB-1691, SK-N-SH, SHEP1 and SHEP/21N/Tet-) were wild-type (wt) p53, while LA1-55N was p53-null. Mouse embryonic fibroblast (MEF) served as control. We obtained all NB lines from H. Findley (Emory University). The SHEP/21N/Tet- was kindly provided by M. Schwab (German Cancer Research Center DFKZ, Heidelberg, Germany). The MEF and 293T cell lines were purchased from the American Type Tissue Collection (ATCC, Bethesda, MD). The 293-T cell line was used for gene transfection assays. The phenotypes of all NB cell lines, including MYCN and p53 status, were confirmed as identical to those published in prior publications (15, 22, 23).

The MX25-1, synthesized in Li lab was dissolved in DMSO to create a 10 mM stock solution, which was stored in small aliquots at -20^0^C. To treat cells, they were exposed to 1-20 µM of MX25-1 for the time period indicated, with the final DMSO concentration kept constant in each experiment. The siMYCN (sc-36003, purchased from Santa Cruz) and miRNA let-7 inhibitor (human antisense-let-7a (has-let-7), purchased from Sigma), were added to the MX25-1 treatment to measure their effects on the sensitivity of cells to MX25-1.

### Plasmid and transfection

The *MYCN* 3’UTR expression plasmid was constructed by inserting the *MYCN* 3’UTR (using primers F: 5’-AAAGGATCCCGCTTCTCAAAACTGGACAG-3’, R: 5’- GCCAAGCTTTAATTTTAAGCTATTTATTT-3’ to amplify the 1697 to 2600 region of the MYCN gene) into the pRNA-CMV3.1-puro vector (Genscript, Piscataway, NJ). The pGL3-*MYCN* 3’UTR reporter plasmid was constructed by inserting the *MYCN* 3’UTR into the pGL3-Promoter (pGL3P) vector. The sense and antisense orientations of the *MYCN* 3’UTR in the luciferase reporter were identified by both enzyme digestion and DNA sequencing. The pRL-CMV vector was co-transfected with the pGL3-*MYCN* 3’UTR plasmid to provide an internal control. Transfection was performed in 6-well plates, using Lipofectamine™ 2000 reagents (Invitrogen) according to the manufacturer’s instructions. Luciferase activity of pGL3-*MYCN* 3’UTR was detected using the Dual-Luciferase Reporter System (Promega).

### Western blot assay

Cell proteins were prepared by lysing cells for 30 min at 4^0^ C in a lysis buffer composed of 150 mM NaCl, 50 mM Tris (pH 8.0), 5 mM EDTA, 1% (v/v) Nonidet p-40, 1 mM phenylmethylsulfonyl fluoride (PMSF), 20 μg/ml aprotinin and 25 μg/ml leupeptin. Equal amounts of protein extracts were resolved by sodium dodecyl sulfate-polyacrylamide gel electrophoresis (SDS-PAGE) and transferred to a nitrocellulose filter. After blocking with buffer containing 5% non-fat milk, 20 mM Tris-HCl (pH 7.5), and 500 mM NaCl for 1 h at room temperature, the filter was incubated with specific antibodies for 1 h at room temperature; washed and then incubated with HRP-labeled secondary antibody; and developed using a chemiluminescent detection system (ECL, Amersham Life Science, Buckinghamshire, England). The specific antibodies used included MYCN (sc-53993, Santa Cruz), MDM2 (SMP14, Sigma; 2A10, Invitrogen), p53 (DO-1, Santa Cruz), HuD (AB5971, Sigma), ARID3B (A06737, Boster) and HMGA2 (PA5-25276, Invitrogen). All antibodies were used according to the manufacturers’ instruction.

### Quantitative RT-PCR

Total RNA was extracted from cells using the RNeasy Mini Kit (Qiagen). First-strand cDNA synthesis was performed with a mixture of random monomers and oligo-dT as primers. Amplification was performed with a 7500 Real-Time PCR System (Applied Biosystems), using the QuantiFast SYBR Green RTPCR kit (Qiagen). All specific primers for amplification of specific genes, as well as the housekeeper gene GAPDH, were purchased from Qiagen. For testing the levels of miRNA let-7 and the internal reference gene RNU24, we use the TagMan-MicroRNA assay kit (ID#: 000377, 000395 and 001001) from Applied Biosystems, according to the manufacturer’s instructions. To specifically amplify and detect expression of MYCN, DICR1, ARID3B and HMGA2, we used the following primer pairs: MYCN (forward (F): 5’-TGCAAGAACCCAGACCTCG-3’, reverse (R): 5’-AGCAGCATCTCCGTGACCC-3’); DICER1 (F: 5’-GTGGACCATTTACTGACA-3’, R: 5’-GAACTACCAATACGGCAC-3’); ARID3B (F: 5’-GAGGAAGAGGACGGAGGT-3’, R: 5’-GGTGCTGGAAGTAGATTGG-3’); HMGA2 (F: 5’-CACTTCAGCCCAGGGACA-3’, R: 5’-CAGTGGCTTCTGCTTTCTTT-3’).

### Compound-RNA and protein-RNA binding assays

We performed fluorescence titration and isothermal titration calorimetry (ITC) assays to determine whether MX25-1 binds to the *MYCN* 3’UTR. For fluorescence titration, *MYCN* 3’UTR was synthesized by *in vitro* transcription with T7 polymerase (MAXIScript T7 RNA polymerase kit, Ambion), and labeled with fluorescence at its 5’end using the 5’ EndTag Nucleic Acid Labeling System (Vector Laboratories, Burlingame, CA, USA). Titration was performed using PTI Quanta-Master spectrometer (Photon Technology International, Birmingham, NJ). The steady-state fluorescence of the RNA-compound mixtures was acquired using a 3 ml cuvette. The slit widths for excitation and emission were adjusted to minimize photobleaching of the sample, while achieving sufficient fluorescent signal intensity. The fluorescence measurements, as a function of reagent concentration, were fitted with the hyperbolic function F = F_f_ + (F_b_ − F_f_)[ligand_f_]/(K_d_ +[ligand_f_]), where F is the observed fluorescence, F_f_ is the fluorescence of unbound protein or RNA, F_b_ is the fluorescence from the RNA-compound complex, ligand_f_ is the concentration of the compound, and K_d_ is its dissociation constant.

ITC assay was performed using the auto-iTC200 instrument (MicroCal, GE). The synthesized *MYCN 3’UTR mRNA* was loaded into a 96 DeepWell PP plate and titrated stepwise into the RNA sample cell using a syringe, for a total of 16 injections (except for the first injection, which was 0.4 μl). The equilibrium time between two adjacent injections was 210 s. The binding stoichiometry (n), binding constant (K_d_), and thermodynamic parameters (ΔH and ΔS) were determined by fitting the titration curve to a one-site binding mode, using the Origin software provided by the manufacturer.

UV cross-linking and immunoprecipitation assays were performed as described previously (15). Briefly, internally labeled *MYCN* 3’UTR probe was synthesized, as described above, in the presence of [α-^32^P] UTP. Cell extracts of NB-1643 treated with or without MX25-1 were immunoprecipitated with MDM2 antibody. The immunoprecipitates were mixed with ^32^P-labeled *MYCN* 3’UTR probes and UV cross-linking of the RNA-protein complexes performed, followed by resolution on 10% SDS-PAGE gels and visualization by autoradiography.

### CHX-chase assay

We performed a standard protein-synthesis inhibitor (CHX) chase assay to measure protein turnover of MYCN and MDM2 in NB-1643 cells treated with MX25-1. Briefly, cells were treated with 50μg/mL CHX for different times before lysis in the presence or absence of MX25-1. Cell lysates were then tested by Western blot analysis to reveal concurrent expression levels of MDM2 and MYCN. The mRNA degradation rate was examined using a standard actinomycin D analysis: At different times after addition of 5 µg/mL of actinomycin D, in the presence or absence of MX25-1, the cells were harvested and their total RNA isolated. The *MYCN* mRNA was detected by quantitative RT-PCR, as described above.

### Clonogenic assay

A clonogenic assay to measure colony formation was used according to a previously described method (24). Briefly, cells were harvested by trypsinization, producing a single-cell suspension, and 200 cells were seeded into a 6-well plate and cultured for 2 weeks. The colonies were stained with a mixture of 6.0% glutaraldehyde and 0.5% crystal violet for about 30 min, carefully removed and rinsed with tap water and counted.

### WST analysis

The cytotoxic effect of MX25-1 on NB cells was determined using the water-soluble tetrazolium salt (WST) assay. Briefly, cells cultured in 96-well microtiter plates were given different concentrations of MX25-1, for a 20-h period. Following this, WST (25 μg/well) was added and incubation continued for an additional 4 h, followed by determination of optical density (OD) with a microplate reader (set at a test wavelength of 450 nm and a reference wavelength of 620 nm). Appropriate controls lacking cells were included to determine background absorbance. WST assay was also used to measure cell growth rate of *MYCN* 3’UTR transfected cells. Briefly, an equal number of *MYCN* 3’UTR-transfected or control cells were seeded in seven microtiter plates and cultured for continuous 1-7 days. WST was applied for 4 h each day in consecutive plates before the OD was read.

### Flow cytometry

Flow cytometry was performed for quantitative detection of apoptosis. Cells with or without treatment were washed with PBS and stained with FITC-annexin V and 7-aminoactinomycin (7-AAD) using a FITC Annexin V Apoptosis Detection Kit 1 (BD Pharmingen™) following manufacture’s instruction and analyzed by flow cytometry.

### Statistical analysis

All data represent mean ± SD of three independent experiments. A two-tailed t-test was performed to compare the difference between two groups. A p value <0.05 is considered significantly different and p>0.5 is considered not significant.

## Results

### MX25-1 inhibits MYCN expression through inducing mRNA degradation in MYCN-amplified NB

**Figure 1:**
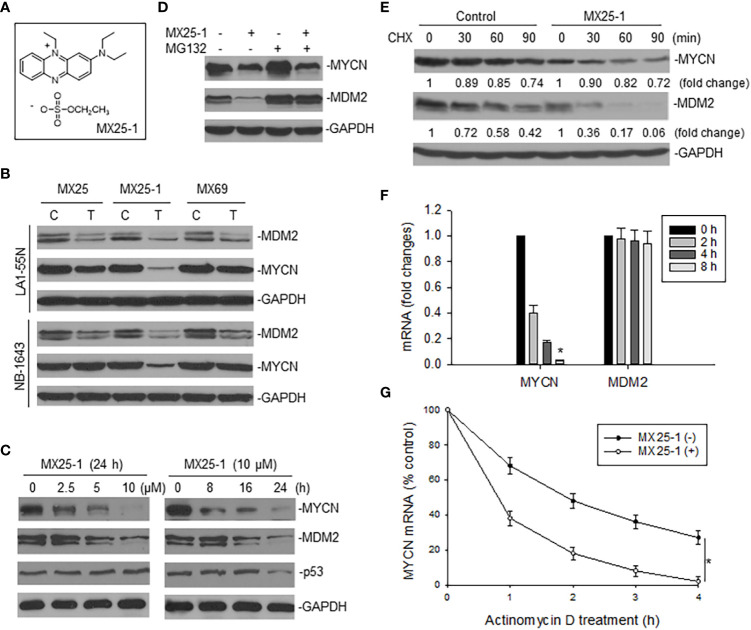
Effect of MX25-1 on MYCN expression in MYCN-amplified NB. **(A)**, structure of MX25-1. **(B)**, Western blot assay for expression of proteins as indicated in NB-1643 and LA1-55N cells treated with 10 µM MX25, MX25-1 and MX69 for 24 h. *C*, control; *T*, treatment. **(C)**, Western blot for dose-response (left) and time-course (7) of MYCN and MDM2 inhibition in NB-1643 cells treated with MX25-1 using indicated conditions. **(D)**, NB-1643 cells with or without MX25-1 treatment (10 µM for 8 h) were treated with 10 µM MG132 for additional 4 h and protein expression then detected by Western blot. **(E)**, protein turnover in NB-1643 cells treated with 10 µM MX25-1 and control for 4 h, as detected by CHX pulse-chase assay. **(F)**, NB-1643 cells were treated with 10 μM MX25-1 for different times, as indicated. The mRNA levels of MYCN and MDM2 relative to GAPDH were determined by qRT-PCR. Data represent mean ± SD of three independent experiments, bars ± SD, *p<0.01. **(G)**, NB-1643 cells were treated with or without 10 µM MX25-1 for 4 h, followed by addition of 5 µg/ml actinomycin D and then harvested at the indicated time points. MYCN mRNA was determined by qRT-PCR, *p<0.05.

We examined the effect of MX25-1 (**Figure 1A**) on expression of MYCN as well as MDM2 in two *MYCN*-amplified NB cell lines NB-1643 (wt-p53) and LA1-55N (p53-null) and found that MX25-1 inhibited the expression of both proteins (**Figure 1B**). As controls, no significant inhibition of MYCN was detected following treatment with either MX25 or MX69, although both compounds were able to inhibit MDM2 expression as previously reported (22). MX25-1 inhibited MYCN and MDM2 expression in a dose- and time-dependent manner (**Figure 1C**). As also shown in **Figure 1C**, levels of p53 expression were not changed in MX25-1-treated NB-1643 cells.

We next evaluated the mechanisms by which MX25-1 inhibits MYCN and MDM2 expression. We first performed MG132 (protein-degradation inhibitor) treatment and cycloheximide (CHX) pulse-chase assay. Results showed that MX25-1 did not induce MYCN protein degradation, while it did induce MDM2 protein degradation. As shown in **Figure 1D**, while MX25-1-mediated downregulation of MDM2 protein was inhibited by MG132, MX25-1-mediated downregulation of MYCN protein was not. CHX pulse-chase assay confirmed that the half-life of MDM2 protein was significantly reduced by MX25-1, whereas the half-life of MYCN protein was not changed in cells similarly treated with MX25-1 (**Figure 1E**).

Next, we tested the effect of MX25-1 on expression and stability of *MYCN* mRNA. Quantitative RT-PCR results showed that MX25-1 significantly reduced the expression and stability of *MYCN* mRNA in NB-1643 cells (**Figures 1F, G**). In contrast, MX25-1 did not alter the *MDM2* mRNA level in this wt-p53 cell line.

### MX25-1 binds to *MYCN* 3’UTR and blocks its interaction with MDM2

**Figure 2:**
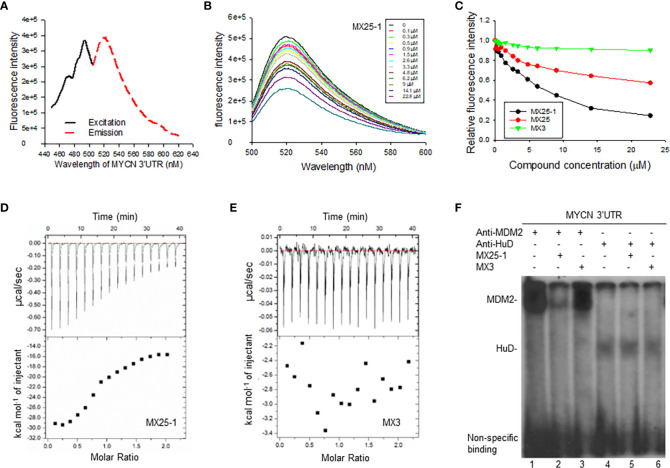
Determination of MX25-1 binding to MYCN 3’-UTR. **(A)**, Excitation and emission spectra of fluorescence-labeled *MYCN 3’UTR*. **(B)**, representative graphs of fluorescence titration assays showing fluorescence changes when MYCN 3’UTR was titrated with MX25-1. **(C)**, MYCN 3’UTR was subject to titration with increasing concentrations of compounds as indicated. Relative changes in fluorescence intensity were traced, as in **(B)**, and normalized to derive a binding K_d_. **(D, E)**, detection of binding between MYCN 3’UTR and MX25-1 **(A)** and non-binding between MYCN 3’UTR and MX3 **(B)** as detected by ITC. Data are representative of three independent experiments. **(F)**, cell extracts from NB-1643 treated with or without MX25-1 or MX3 (as control) were incubated with ^32^P-labeled RNA probes corresponding to the MYCN 3’UTR element, UV cross-linked, and then immunoprecipitated with anti-MDM2 and anti-HuD antibodies. The protein/RNA probe complexes were run on an SDS-PAGE gel and imaged by autoradiography.

Since the 3’UTR of *MYCN* tightly regulates stability of *MYCN* mRNA, we tested whether MX25-1-induced *MYCN* mRNA degradation is through binding to and inhibiting its 3’UTR activity. We performed fluorescence titration assays by labeling *MYCN* 3’UTR with fluorescence having excitation and emission wavelengths of 485 nm and 530 nm, respectively (**Figure 2A**), followed by titrating with MX25-1; MX25 and MX3 were used as controls. We found that MX25-1 bound to *MYCN* 3’UTR as demonstrated by the significant decrease in fluorescence intensity with increasing MX25-1 concentrations (**Figure 2B**); fluorescence intensity was quantitated and normalized to derive a binding K_d_ for MX25-1 of 7.2 µM (**Figure 2C**). MX25 bound to *MYCN* 3’UTR with a much lower affinity (K_d_ of 26.8) µM), while MX3 did not bind at all, although both MX25 and MX3 bound to the *XIAP* IRES mRNA (22). We also performed ITC assays to confirm binding of MX25-1 to *MYCN* 3’UTR. Consistent with the fluorescent titration results, MX25-1 (but not MX3) bound to the *MYCN* 3’UTR with a binding K_d_ of 6.75 uM (**Figures 2D, E**).

We have previously reported that *MYCN* 3’UTR interacted with MDM2 protein and stabilized *MYCN* mRNA, accompanied by an increase in MYCN translational activity (15). Here, we tested whether binding of MX25-1 to *MYCN* 3’UTR was able to block the interaction between MDM2 protein and *MYCN* 3’UTR mRNA, thereby destabilizing *MYCN* mRNA. We performed protein-RNA binding assays by UV cross-linking for a ^32^P-labeled *MYCN* 3’UTR probe and cell extracts from NB-1643 cells, followed by immunoprecipitation with MDM2 and HuD (as control) antibodies. Consistent with previously reported results, both MDM2 and HuD were able to bind to the *MYCN* 3’UTR probe (15). Addition of MX25-1 significantly reduced the formation of MDM2-*MYCN* 3’UTR probe complex (**Figure 2F**). MX25-1 did not affect the formation of HuD-*MYCN* 3’UTR, and MX3 affected neither MDM2-*MYCN* 3’UTR nor HuD-*MYCN* 3’UTR interaction.

### MX25-1 inhibits *MYCN* 3’UTR activity resulting in activation of let-7

**Figure 3:**
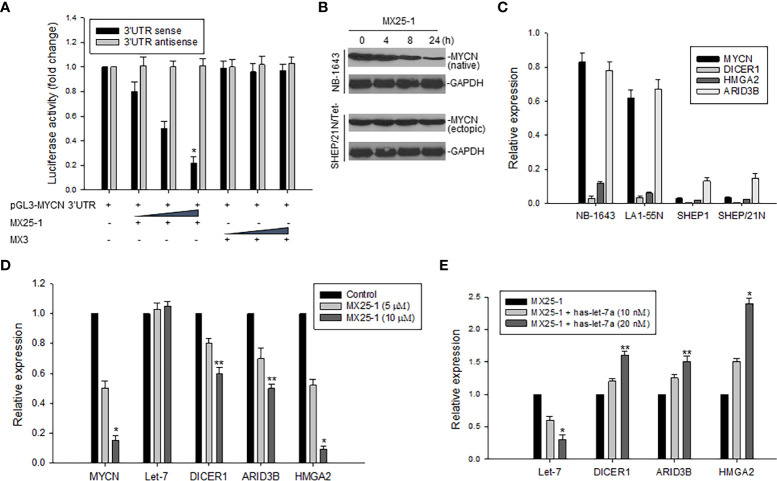
Effect of MX25-1 on MYCN 3’UTR activity. **(A)**, 293T cells were transfected with 5μg pGL3-MYCN 3’UTR sense or antisense (as control) plasmids and treated with increasing amounts (2.5, 5, and 10 μg) of MX25-1 or control compound. Luciferase activity of pGL3-MYCN 3’UTR was detected using the Dual-Luciferase Reporter System, *p<0.01. **(B)**, cell lines (NB-1643 with MYCN gene amplification and SHEP/21N/Tet- with conditional MYCN expression) were treated with 10 µM MX25-1 for different times, and the expression of MYCN (native) in NB-1643 and MYCN (ectopic) in SHEP/21N/Tet- were detected by Western blot assay. **(C)**, expression of mRNA for indicated genes in 4 NB cell lines with or without *MYCN* amplification, as detected by qRT-PCR. Results are normalized to the internal control GAPDH. **(D)**, expression of mRNA of MYCN, DICER1, ARID3B and HMGA2 as well as miRNA let-7 in NB-1643 cells treated with MX25-1 at indicated doses for 24 h, as detected by quantitative RT-PCR. Results of let-7 are normalized to the internal control RUN24, *p<0.01, **p<0.05. **(E)**, similar quantitative RT-PCR assays for mRNA and miRNA expression of indicated genes in NB-1643 cells treated with MX25-1 (10 µM for 24 h) in the absence and presence of the let-7 inhibitor (has-let-7a), *p<0.01, **p<0.05. Data in panels **(A, C–E)** represent mean of three independent experiments, bars ± SD.

We constructed a luciferase reporter plasmid in a pGL3-promoter containing the *MYCN* 3’UTR and performed gene transfection and reporter assays. Results as shown in **Figure 3A** indicate that MX25-1 significantly reduced *MYCN* 3’UTR sense-mediated (but not antisense-mediated) luciferase activity, suggesting that MX25-1 destabilizes *MYCN* mRNA by regulating the ARE within *MYCN* 3’UTR. Furthermore, the endogenous *MYCN* gene containing the *MYCN* 3’UTR sequence (in NB-1643 cells) was inhibited by MX25-1; in contrast, the transfected *MYCN* gene lacking the *MYCN* 3’UTR sequence (in SHEP/21N/Tet- cells) was not inhibited by MX25-1 (**Figure 3B**), confirming the effect of MX25-1 on *MYCN* 3’UTR activity.

In addition, we evaluated the effect of MX25-1-mediated downregulation of *MYCN* 3’UTR activity on its downstream miRNA let-7 and the let-7 targets (*DICER1, ARID3B* and *HMGA2*). First, we performed qRT-PCR to detect expression of mRNA and found that *MYCN*-amplified NB-1643 and LA1-55N cells expressed higher levels of *DICER1, ARID3B* and *HMGA2* than the non *MYCN*-amplified SHEP1 and SHEP/21N/Tet- cells (**Figure 3C**). We then treated NB-1643 cells with MX25-1. Although the change of let-7 expression was not obvious following marked downregulation of *MYCN* mRNA, the expression of *DICER1, ARID3B* and *HMGA2* was significantly reduced (**Figure 3D**). We believe that inhibition of these let-7 targets is due to release of let-7 from the *MYCN* mRNA/let-7 complex following MX25-1-mediated degradation of *MYCN* mRNA, resulting in increased let-7 activity.

To confirm that the reduced expression of *DICR1, ARID3B* and *HMGA2* in MX25-1-treated cells is indeed a result of let-7 activation following MX25-1-induced *MYCN* mRNA degradation, we performed similar MX25-1 treatment and qRT-PCR analysis as in **Figure 3D** in the presence of let-7 inhibitor (has-let-7a). Inhibition of let-7 resulted in increased expression of these let-7 targets (**Figure 3E**).

### MX25-1 potently induces cell growth inhibition and death in *MYCN*-amplified NB cells

**Figure 4:**
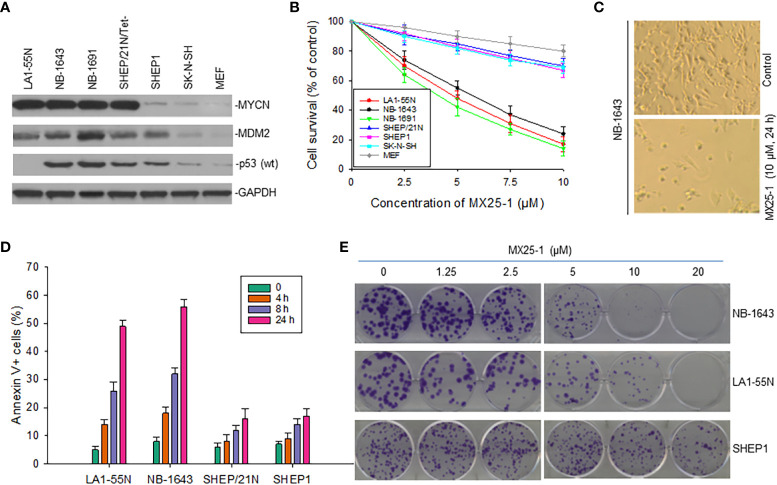
Inhibitory and cytotoxic effects of MX25-1 on NB cells with and without MYCN overexpression. **(A)**, Western blot assay for protein expression of MYCN, MDM2 and p53 in six cultured NB cell lines (LA1-5S,NB-1643 and NB-1691 with MYCN amplification, SK-N-SH and SHEP1 without MYCN amplification, SHEP/21N/Tet- with conditional MYCN). Mouse embryonic fibroblast (MEF) served as control. **(B)**, WST assay for cytotoxic effect of MX25-1 on these lines. Cells were treated with indicated doses of MX25-1 for 24 h. Data represent mean of three independent experiments. **(C)**, representative micrographs showing inhibition of NB-1643 cells treated with MX25-1 as compared to control. **(D)**, apoptosis following treatment of above cell lines with 10 µM MX25-1 for the indicated times; apoptotic cells were quantitated by flow cytometry. **(E)**, representative colony formation of above lines treated with or without MX25-1 for two weeks.

Since MX25-1 targets *MYCN* 3’UTR for mRNA degradation, we evaluated the effect of this compound on the viability and growth of six NB cell lines with or without *MYCN* gene amplification/overexpression (**Figure 4A**). By WST cytotoxicity assay, MX25-1 exhibited potent cytotoxic activity for *MYCN*-amplified NB-1643,LA1-55N and NB-1691 cells. MX25-1 showed less cytotoxicity for either SK-N-SH and SHEP1 lacking *MYCN* amplification or SHEP/21N/Tet- with conditional expression of MYCN as well as the non-tumorigenic cells MEF (**Figures 4B, C**).

Additionally, we tested whether MX25-1-induced cell death occurs by apoptosis. We stained cells with Annexin-V FITC and 7-ADD and quantitated the results by flow cytometry. Most cells were found to be Annexin-V positive, indicating apoptosis early in the treatment (after 4-8 h of MX25-1). Consistent with the WST cytotoxicity results, MX25-1 induced strong apoptosis in NB-1643 and LA1-55N, but much less apoptosis in SHEP1 and SHEP/21N/Tet- (**Figure 4D**).

Furthermore, results of colony formation assays showed that MX25-1 potently inhibited NB-1643 and LA1-55N cell growth. We observed a significant reduction in both colony number and size in MX25-1-treated NB-1643 and LA1-55N cells, as compared with the control (**Figure 4E**). In contrast, MX25-1 had much less inhibitory effect on SHEP1 colony formation.

### *MYCN* 3’UTR expression and let-7 activation are critical in MX25-1-induced cell growth inhibition and death

**Figure 5:**
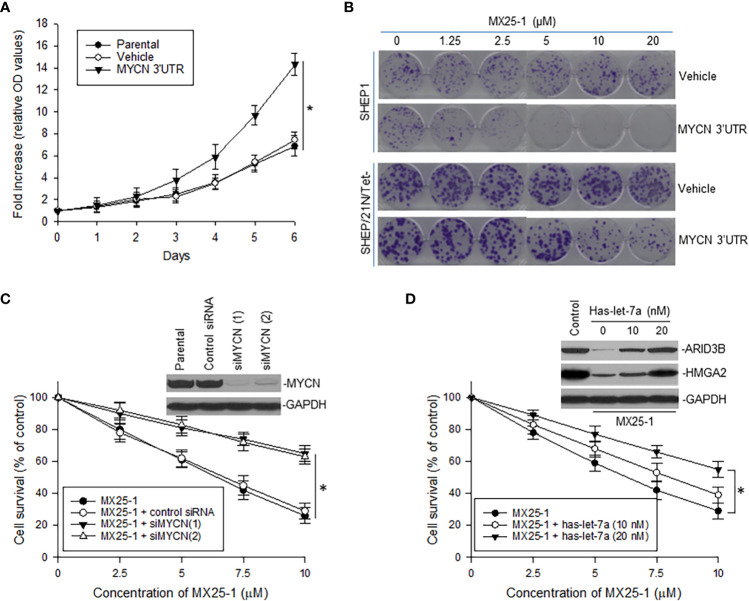
Effect of MYCN 3’UTR-regulated signals on NB cell growth and sensitivity to MX25-1. **(A)**, WST proliferation assay comparing the growth of parental SHEP1 and SHEP1 transfected with plasmids as indicated. *p<0.01. **(B)**, representative colony formation by above cell lines transfected with either MYCN 3’UTR or vehicle control; cells were treated with or without MX25-1 for two weeks. **(C)**, NB-1643 cells transfected with siMYCN were treated with MX25-1 and cell survival detected by WST assays, *p<0.01; Insert, MYCN expression as detected by Western blot. **(D)**, WST results show cell survival of NB-1643 following treatment with MX25-1 in the presence or absence of let-7 inhibitor (Has-let-7a), *p<0.05; Insert, expression of let-7 targets ARID3B and HMGA2 as detected by Western blot. Data in panels **(A, C, D)** represent mean ± SD of three independent experiments.

To evaluate the role of *MYCN* 3’UTR in regulating NB cell growth and sensitivity to MX25-1, we transfected non-*MYCN*-amplified SHEP1 cells with a plasmid conditionally expressing *MYCN* 3’UTR. Overexpression of *MYCN* 3’UTR significantly stimulated cell proliferation (**Figure 5A**). When treated with MX25-1, colony formation by *MYCN* 3’UTR-transfected SHEP1 cells was significantly inhibited as compared with the same cells transfected with control (**Figure 5B**, upper panel). Enforced expression of *MYCN* 3’UTR in SHEP/21N/Tet- (overexpressing MYCN protein but lacking MYCN 3’UTR) also inhibited cell growth and colony formation (**Figure 5B**, lower panel).

In contrast, we performed gene knockout (KO) of MYCN by siRNA in NB-1643 cells and then treated these cells with MX25-1. Inhibition of *MYCN* mRNA expression significantly reduced sensitivity of these cells to MX25-1 (**Figure 5C**), further confirming that MX25-1 targets *MYCN* mRNA and that MX25-1-induced cell death is *MYCN* mRNA dependent.

Furthermore, we performed cytotoxicity assays with NB-1643 cells treated with MX25-1 in the presence of the let-7 inhibiter has-let-7a. Inhibition of let-7 also decreased sensitivity of these cells to MX25-1 (**Figure 5D**). We observed a reduced expression of the let-7 targets ARID3B and HMGA2 in MX25-1-treated cells in the absence of has-let-7a, and a recovered expression of these proteins when has-let-7a was added (**Figure 5D**, insert). These results suggest that *MYCN* mRNA-let-7 mediated signals play an important role in MX25-1-induced inhibition and death in *MYCN*-amplified NB.

## Discussion

In this study, we have identified a small-molecule compound MX25-1 as a novel and potent regulator of *MYCN* 3’UTR mRNA. We demonstrated that MX25-1 bound to the 3’UTR of *MYCN* mRNA leading to inhibition of its activity. Downregulation of *MYCN* 3’UTR activity induced by MX25-1 resulted in mRNA degradation and activation of miRNA let-7 accompanied by marked inhibition of cell growth and potent cell death; this inhibition was specific for *MYCN*-amplified and *MYCN* 3’UTR overexpressing NB cells.

Due to the significant role of MYCN in NB and some other tumor types, together with the fact that it is overexpressed in tumors but not in normal tissue, MYCN represents a good therapeutic target. However, as in the case of other transcription factors, a MYCN-specific therapeutic agent has not yet been identified. This is not only because of the complex roles that MYCN plays in various aspects of cellular physiology, but also the fact that MYCN functions are largely mediated through protein-protein interactions; thus, it is difficult to design specific small molecules to block these large surface interactions.

Theoretically, inhibition of MYCN expression can be achieved by targeting its mRNA through either suppressing its transcription or inducing its degradation. For instance, retinoic acid is known to inhibit *MYCN* mRNA transcription, and this compound is currently used for therapy of *MYCN*-amplified NB (25, 26). Similarly, knockout of MYCN expression with either antisense or siRNA has been reported to decrease tumorigenesis and increase sensitivity of *MYCN*-amplified NB cells to anticancer drugs (27, 28).

In an effort to target *MYCN* mRNA degradation, we derived the small-molecule compound MX25-1 from a previously published compound MX25 (22). The latter compound was originally selected to bind to the IRES mRNA of *XIAP* and block the interaction between *XIAP* IRES and MDM2, resulting in inhibition of both MDM2 and XIAP (22). In an attempt to increase the anticancer activity of MX25 as a potential candidate for drug development, we performed focused chemical modification and screened a panel of MX25 analogs. We found one derivative (MX25-1, containing ethyl sulfate as the counter ion) that showed increased cytotoxicity specifically for *MYCN*-amplified NB. Further studies showed that MX25-1 bound to *MYCN* 3’UTR mRNA and inhibited its activity, leading to mRNA degradation.

**Figure 6:**
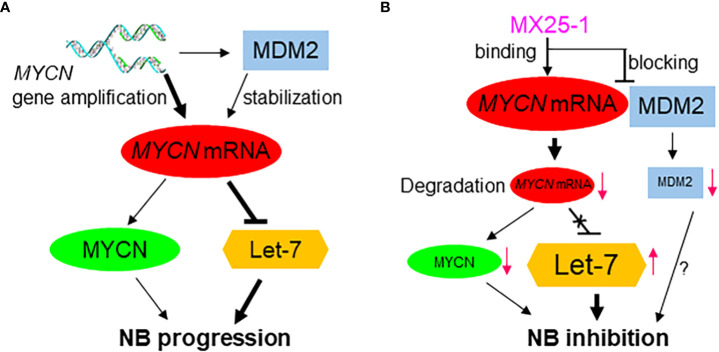
Proposed model for the role of high-level MYCN mRNA in MYCN-amplified NB progression, showing MYCN mRNA exerts a MYCN-protein-independent effect by inhibiting the tumor-suppressor miRNA Let-7 and inducing tumor progression **(A)**; Effect of MX25-1 in blocking MYCN mRNA-MDM2 interaction, resulting in degradation of MYCN mRNA and activation of Let-7 in MYCN-amplified NB **(B)**.

We have previously reported that the stabilization of *MYCN* mRNA is regulated by MDM2 through binding of the MDM2 C-terminal RING domain to the *MYCN* 3’UTR (**Figure 6A**) (15). Thus, we hypothesized that binding of MX25-1 to *MYCN* 3’UTR could potentially block or disrupt the interaction between *MYCN* 3’UTR and MDM2 RING domain (**Figure 6B**). Our results support this hypothesis and show that treatment with MX25-1 results in activation of AU-rich elements (ARE) within the *MYCN* 3’UTR, leading to rapid degradation of the mRNA. However, we cannot rule out that binding of MX25-1 to *MYCN* 3’UTR may promote *MYCN* mRNA degradation through additional mechanisms. Our results further show that, in addition to destabilizing *MYCN* mRNA, MX25-1 treatment results in degradation of MDM2 protein, which is also likely due to MX25-1-mediated disruption of MDM2-*MYCN* mRNA complexes; we have observed similar degradation of MDM2 in MX25-mediated disruption of MDM2-*XIAP* IRES complexes (22).

It has been reported that overexpression of *MYCN* 3’UTR mRNA in *MYCN*-amplified NB has an independent function in promoting NB cell proliferation. Our experimental results in this study support this notion. First, we performed transfection a *MYCN* 3’UTR expression plasmid into SHEP1 cells lacking *MYCN*-amplification. Enforced overexpression of the *MYCN* 3’UTR promoted cell growth and sensitized these cells to MX25-1-induced inhibition. Furthermore, transfection of *MYCN* 3’UTR into SHEP/21N/Tet- cells, which conditionally overexpress MYCN protein lacking the *MYCN* 3’UTR, also enhanced sensitivity of these cells to MX25-1. These results indicate an independent role for *MYCN* 3’UTR beyond that of MYCN protein in promoting NB and suggest that inhibition of *MYCN* 3’UTR activity contributes to MX25-1-induced cell growth inhibition and apoptosis.

The critical role of *MYCN* 3’UTR in promoting NB cell growth, and the ability of MX25-1 to downregulate its activity was further confirmed by our studies of miRNA let-7. Let-7 serves as a potent tumor suppressor through post-transcriptional repression of a number of oncogenic mRNA targets including *DICR1, ARID3B, HMGA2* and *MYCN* (8–10, 29). In the case of MYCN, high-levels of *MYCN* 3’UTR transcribed from the amplified *MYCN* gene bind to and sequester let-7, resulting in inhibition of its activity (**Figure 6A**) (7). By examining the expression of DICR1, ARID3B and HMGA2 in NB cell lines, we found that high-level expression of these genes is indeed associated with *MYCN* gene amplification. Conversely, degradation of *MYCN* mRNA *via* binding of MX25-1 to *MYCN* 3’UTR was accompanied by reduced expression of DICR1, ARID3B and HMGA2. In contrast, antisense-mediated inhibition of let-7 expression increased expression of these oncogenic targets in MX25-1-treated cells and rescued them from growth inhibition and death.

Although MX25-1 also induced degradation of MDM2 oncoprotein in *MYCN*-amplified NB, the MDM2-p53 pathway appears dispensable for the cytotoxic activity of MX25-1 in this type of cancer. Our results showed that MX25-1 induced similarly potent cell growth inhibition and death in *MYCN*-amplified cells with either p53-null or wt-p53 phenotypes (e.g. LA1-55N and NB-1643 cells, respectively), suggesting a p53-independent mechanism for MX25-1 activity. In fact, Western blot results showed no changes in p53 expression in MX25-1-treated NB-1643 cells (**Figure 1C**). The absence of p53 activation after MX25-1-mediated degradation of MDM2 is consistent with siRNA-mediated knockout of MDM2 in *MYCN*-amplified NB cells, which also fails to activate p53, as reported in our previous publication (21). This might be due to mutual regulation among MYCN, MDM2, and p53: MYCN stimulates MDM2 and p53 transcription (3, 5), whereas MDM2 stabilizes *MYCN* mRNA (15). In MX25-1 or siMDM2-treated *MYCN*-amplified cells, although p53 protein is stabilized due to inhibition of MDM2, p53 transcription is inhibited by downregulation of MYCN. Therefore, p53 expression and activity remained unchanged.

Due to the difficulty in pharmacologically targeting MYCN protein, along with the independent role of *MYCN* mRNA in regulating NB progression, targeting of *MYCN* mRNA by small-molecule inhibitors may represent an attractive strategy for therapy of *MYCN*-amplified NB. Such therapy would both inhibit MYCN’s cell-survival effects and activate the tumor-suppressor effect of let-7 (**Figure 6B**). We have found that MX25-1 exhibits these effects by binding to *MYCN* 3’UTR and inducing *MYCN* mRNA degradation, thus providing proof-of-principle that targeting *MYCN* mRNA is likely an effective and novel therapeutic approach for *MYCN*-amplified/refractory NB patients.

## Data availability statement

The raw data supporting the conclusions of this article will be made available by the authors, without undue reservation.

## Author contributions

TL and LG carried out the experiment. ZW and NA performed organic synthesis. MZ conceived the original idea and wrote the manuscript. MZ and WL supervised the project. All authors contributed to the article and approved the submitted version.

## Funding

This work was supported by an R01 grant (CA240447) to MZ and WL; a Research grant (201C40) from Rally Foundation to MZ; and a Scholar Award (713905) from Hyundai Hope on Wheels to MZ.

## Conflict of interest

The authors declare that the research was conducted in the absence of any commercial or financial relationships that could be construed as a potential conflict of interest.

## Publisher’s note

All claims expressed in this article are solely those of the authors and do not necessarily represent those of their affiliated organizations, or those of the publisher, the editors and the reviewers. Any product that may be evaluated in this article, or claim that may be made by its manufacturer, is not guaranteed or endorsed by the publisher.
